# Advances in 3D Bioprinted Cardiac Tissue Using Stem Cell-Derived Cardiomyocytes

**DOI:** 10.1093/stcltm/szae014

**Published:** 2024-03-19

**Authors:** Jacqueline M Bliley, Maria A Stang, Anne Behre, Adam W Feinberg

**Affiliations:** Department of Biomedical Engineering, Carnegie Mellon University, Pittsburgh, PA, USA; Department of Biomedical Engineering, Carnegie Mellon University, Pittsburgh, PA, USA; Department of Biomedical Engineering, Carnegie Mellon University, Pittsburgh, PA, USA; Department of Biomedical Engineering, Carnegie Mellon University, Pittsburgh, PA, USA; Department of Materials Science and Engineering, Carnegie Mellon University, Pittsburgh, PA, USA

**Keywords:** cardiac, tissue engineering, stem cells, myogenesis, differentiation

## Abstract

The ultimate goal of cardiac tissue engineering is to generate new muscle to repair or replace the damaged heart. This requires advances in stem cell technologies to differentiate billions of cardiomyocytes, together with advanced biofabrication approaches such as 3D bioprinting to achieve the requisite structure and contractile function. In this concise review, we cover recent progress in 3D bioprinting of cardiac tissue using pluripotent stem cell-derived cardiomyocytes, key design criteria for engineering aligned cardiac tissues, and ongoing challenges in the field that must be addressed to realize this goal.

## Graphical Abstract

**Graphical Abstract:**
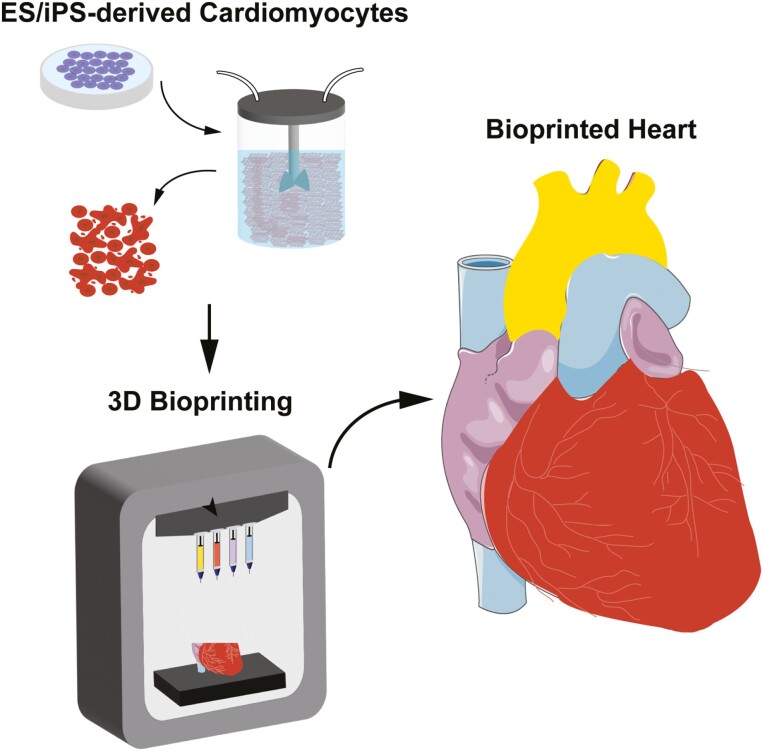

**Significance Statement**

Recent progress in the biofabrication of human cardiac muscle tissue demonstrates the unprecedented advances in structure and function that can be achieved. Despite this, significant barriers to building clinically relevant cardiac tissues remain, including cost, the maturation state of the bioprinted tissues, and the inability to manufacture the quantity and diversity of cell types. Here we discuss recent advances in stem cell technologies to overcome these challenges, such as cell scale-up and maturation via bioreactors, and provide unique insight into what the appropriate building blocks are for generating these tissues on the path toward clinical translation.

## Introduction

The human heart is a unique organ, contracting billions of times during a person’s lifetime and undergoing constant self-renewal at the molecular level to maintain high metabolic activity and physiologic function. However, following injury or disease, the heart is unable to repair itself due to a lack of a resident stem cell population and the terminal differentiation of cardiomyocytes.^1^ In the context of disease this ultimately leads to heart failure, where decreased contractility results in loss of pump function. To address this, pluripotent stem cells have emerged as a key technology, enabling the differentiation of human cardiomyocytes and other cardiac cell types (eg, smooth muscle cells and endothelial cells) with the goal to repair or ultimately replace the whole heart.^2-5^

Engineering cardiac muscle has always proved challenging because terminally differentiated adult cardiomyocytes isolated from heart tissue rapidly dedifferentiate and become senescent, making them unusable. This is where current progress in embryonic stem cell (ESC) and induced pluripotent stem cell (iPSC) differentiation has enabled the generation of multiple cardiac cell types, which has been described extensively elsewhere.^3,6^ However, this is only the first step in engineering functional cardiac tissue. The ability of less mature cardiomyocytes to be used to engineer 3D heart tissue was pioneered by Eschenhagen and coworkers using neonatal rat cardiomyocytes cast in hydrogels that formed aligned cardiac muscle due to cell-generated forces.^7-11^ Since then, this approach has been used widely to build linear strips, sheets, and ventricle-like cups through casting and molding.^12^ While progress has been made, these approaches are limited in the 3D tissue architectures that can be created due to the limited control of cardiomyocyte organization. To address this, decellularization of heart tissue has been pursued as a way to produce extracellular matrix (ECM) scaffolds that recapitulate nearly all of the native 3D architecture. However, it has proved particularly challenging to recellularize these ECM scaffolds with cardiomyocytes because the cells need to be delivered to parenchymal space, resulting in modest cellularity per cross-sectional area of about 10%-35%.^13^ In comparison, 3D bioprinting leverages computer-controlled robotic deposition of cells and biomaterials in a layer-by-layer process to fabricate tissues with precise control of composition and structure and can achieve cellularity approaching that of native myocardium. This has emerged as a disruptive technology to engineer more realistic cardiac tissue, including using computer-aided design (CAD) models based on medical imaging data to create patient-specific tissues and eventually organs. In this concise review, we will use the forward-looking example of building a whole heart to understand how advances in stem cell engineering and 3D bioprinting are converging to enable this new field, and how this can be applied to other tissue and organ systems.

## The Potential of 3D Bioprinting to Rebuild the Human Heart

Rebuilding the human heart requires an understanding of its structure and function, particularly how specific cell populations and their architectural arrangement contribute to contractility. For example, the 4-chambered heart is formed of atria and ventricles with muscular walls separated by valves to ensure unidirectional blood flow. The ventricular walls consist of laminar sheets of cardiac muscle arranged in a helical pattern.^14^ Within these sheets, cardiomyocytes are uniaxially aligned and coupled end-to-end to maximize force production and cardiac output, which ensures efficient shortening and force generation parallel to the longitudinal axis of the layer. Pumping is initiated through a precisely patterned conduction system that starts muscle contraction within cardiomyocytes in a spatially defined 3D sequence. Interestingly, cardiomyocytes and the nodal and Purkinje cells of the conduction systems make up >50% of the cell mass but <30% of the total cell number within the heart. Indeed, there are 11 major cell types within the adult human heart,^15^ including fibroblasts, smooth muscle cells, pericytes, endothelial cells, immune cells, mesothelial cells, and adipocytes. While stem cell differentiation protocols for many of these cell types have already been developed,^16^ differentiation of specific cardiac cell types can be complex and difficult to reproduce. It is still challenging to efficiently generate highly pure cardiac cell types, such as those of the conduction system, like nodal and Purkinje cells.^17-20^ Further, protocols for the generation of endocardial and valvular interstitial cells have only recently been established.^21,22^ Importantly, these stem cell-derived cardiac cell types often exhibit fetal-like phenotypes, making maturation toward adult-like phenotypes an ongoing challenge to achieve improved function.

Three-dimenstional bioprinting is a potential solution to rebuild the complex 3D architecture of the heart because it enables precise positioning of cells and biomaterials in 3D space using robotic control. There are multiple approaches within 3D bioprinting, including light-based stereolithography (SLA) and digital light processing (DLP), and extrusion-based techniques, which each has their own distinct advantages and disadvantages.^23-25^ All approaches enable a CAD model, either designed de novo or based on medical imaging data, such as computed tomography (CT) or magnetic resonance imaging (MRI), to be transformed into a physical object. To do this, the CAD model is computationally sliced into layers that represent a thin cross-section of the 3D model, providing instructions for machine pathing to create the printed structure through printing layer by layer.

**Figure 1.**
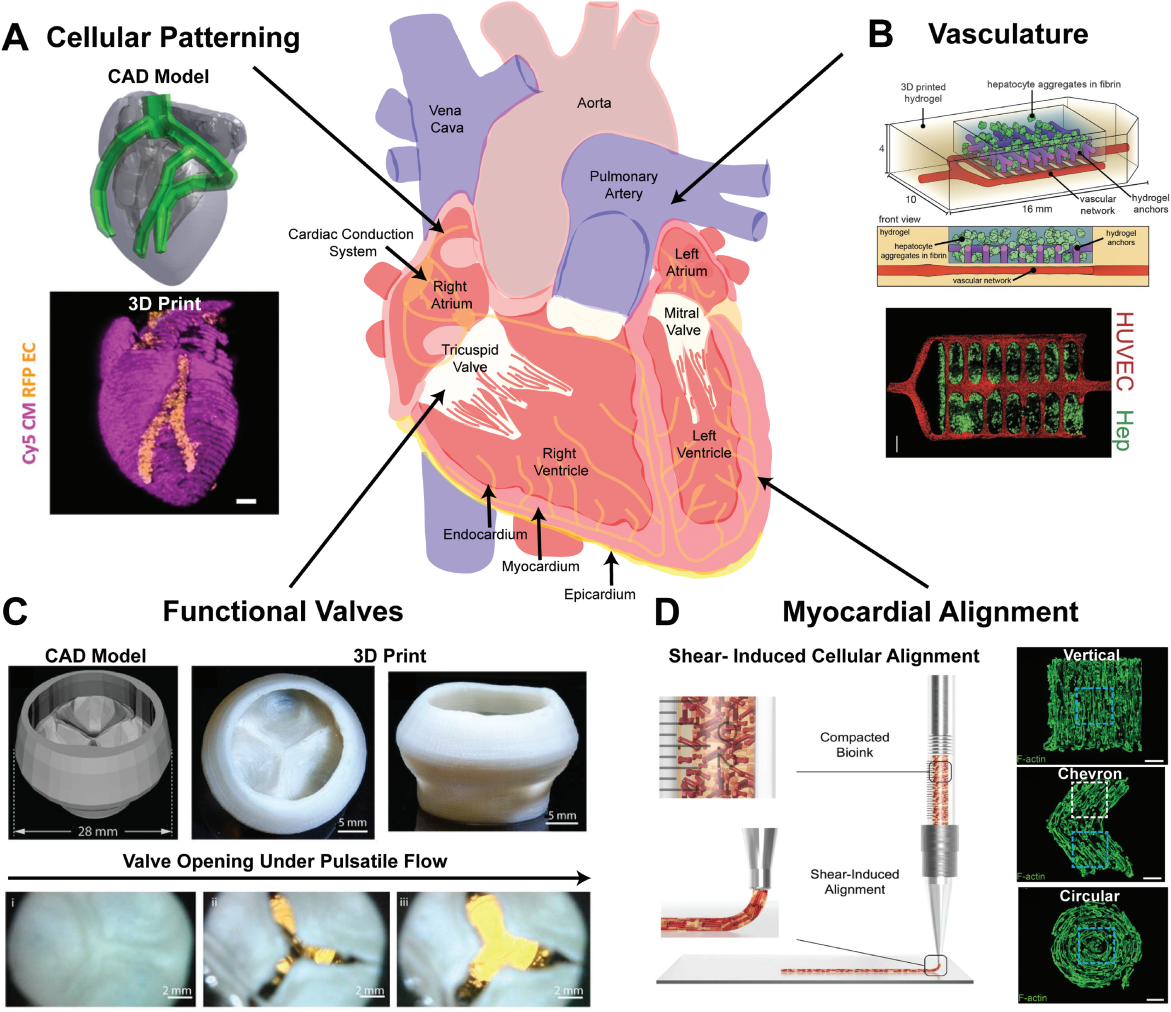
Capabilities of bioprinting to build different features of the heart. Bioprinting has enabled the (**A**) spatial patterning of cells like endothelial cells (red) within a cardiomyocyte structure (pink).^27^ (**B**) Vasculature has been created using light-based bioprinting and subsequently endothelialized (red), forming perfusable channels within hepatocyte aggregates (green).^28^ (**C**) Creation of functional acellular components has been accomplished, including trileaflet heart valves that open and close under physiologically relevant flows and pressures.^5^ (**D**) Reconstruction of microscale structural features, like myocardial alignment, has been accomplished utilizing shear stress through the needle during extrusion-based bioprinting.^29^

The research community has already achieved multiple milestones toward 3D bioprinting a human heart (Fig. 1). First, patient-specific CAD models can be derived from MRI data and then 3D bioprinted directly out of type I collagen, the predominant ECM within the adult human heart.^5^ In contrast to other tissue engineering techniques, such as casting/molding, complex internal components of the heart can be created with 3D bioprinting, such as the internal chambers of the heart and trabeculae, which is a sponge-like network of muscle bundles that protrude from the inner surfaces of the ventricles. To date, full-size bioprinted hearts are not cellularized, but these organ-scale constructs can be used to assess the accuracy compared to the CAD model, a process termed gauging, and provide patient-specific heart phantoms that mimic the native tissue mechanics, which may help with surgical planning.^5,26^ Second, 3D bioprinting can achieve spatial control through deposition of different cellular inks into discrete locations, such as segregation of endothelial cells within the coronary vasculature of the heart and cardiomyocytes within the myocardium (Fig. 1A).^27^ Third, the high metabolic demand of the heart requires vasculature that spans multiple length scales from arteries to capillaries. Vessels can be created with both extrusion based and SLA bioprinting and then endothelialized post-print to obtain perfusable vasculature that is able to support the viability of cells within the printed structure^28^ (Fig. 1B). Fourth, unidirectional flow through the heart and cardiac output requires valves, which have been 3D bioprinted out of collagen and demonstrated the ability to open and close under pulsatile, physiologic pressures and flow rates (Fig. 1C).^5^ Finally, alignment of cardiomyocytes is critically important in obtaining the required contractile function, where shear stress through the needle during extrusion 3D bioprinting can be used to direct the alignment of cardiomyocytes to create uniaxial, chevron, and even circularly aligned cardiac constructs (Fig. 1D). While significant advances have been made, it is important to understand that there are still many more critical aspects of the heart that may be required to build a full organ, such as nodal tissue to regulate heart rate, a cardiac conduction system to control activation patterns, and papillary muscles and chordae tendineae to prevent valve regurgitation.

## State of the Art in the 3D Bioprinting of Cardiac Tissue

**Figure 2.**
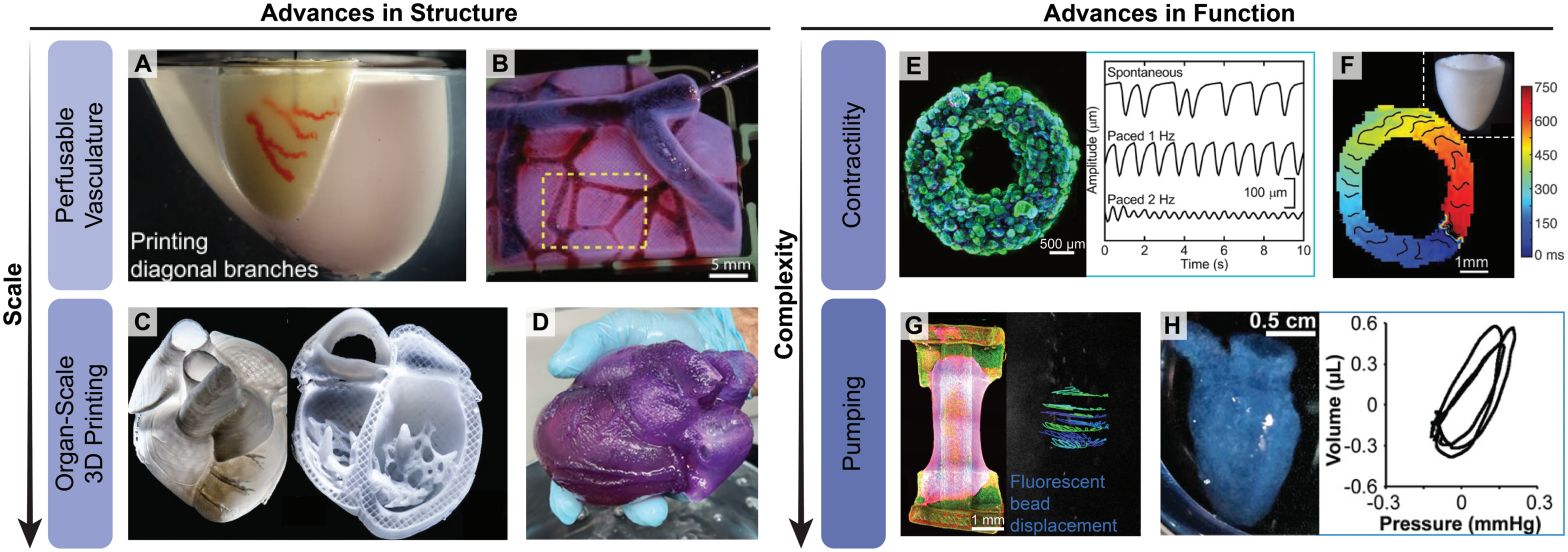
Recent advances in the structure and function of bioprinted cardiac tissues. Bioprinting has enabled the fabrication of biomimetic cardiac features, including (**A**) SWIFT-printed perfusable branching vascular channels within a cardiac tissue matrix consisting of compacted OBBs, ECM, and fibroblasts, as well as structures printed via FRESH, including (**B**) a perfusable, multiscale, interconnected vascular network, printed from collagen type I (scale bar: 5 mm); (**C**) a neonatal-scale human heart with internal features such as trabeculae, printed from collagen type I; and (**D**) an adult-scale human heart printed out of alginate that possesses features like valves and ventricles. Reprinted (adapted) with permission from^26^ (Copyright 2023 American Chemical Society). Bioprinted cardiac tissues also demonstrate improvements in function: (**E**) a SWIFT cardiac tissue demonstrated contractility with sensitivity to electrical stimuli (scale bar = 500 µm); (**F**) a simplified model of the left ventricle FRESH-printed using collagen type I demonstrated circumferential calcium wavefront propagation (scale bar: 1 mm); (**G**) hiPSC-derived cardiomyocytes and fibroblasts cast in a collagen gel around a FRESH-printed collagen linear heart tube induced unidirectional fluorescent bead displacement, indicating pumping capabilities (scale bar: 1 mm); and (**H**) a FRESH-printed hChaMP demonstrated pumping and thus pressure generation, quantified as pressure-volume loops (scale bar = 0.5 cm). This figure is adapted with permission from references.^4,5,26,32,33^

The application of 3D bioprinting to cardiac tissue engineering has enabled the fabrication of architecturally and functionally complex cardiac structures that span across multiple lengths scales, from alignment at the cellular level to valves and ventricles at the organ scale. For example, light-based microscale continuous optical printing (μCOP) can align neonatal mouse ventricular cardiomyocytes into fiber-like structures within cardiac microtissues.^29^ Direct ink writing (DIW), an extrusion-based printing approach, was also used to control cellular alignment in cardiac tissue sheets (~1 cm^2^) in linear, chevron, and spiral architectures (Fig. 1D). To do this, cardiac microtissue strips, referred to as anisotropic organ building blocks (aOBBs, about 2 mm long, 250 µm wide), were generated from human-induced pluripotent stem cell iPSC (hiPSC)-derived cardiomyocytes and formed into a bioink. Upon extrusion, the aOBBs aligned in the print path direction due to shear and extensional forces.^30^ OBBs have also been used in sacrificial writing into functional tissue (SWIFT) bioprinting, where a sacrificial gelatin ink is extruded into a compacted slurry of cardiomyocyte OBBs, ECM, and fibroblasts (Fig. 2A).^31^ Evacuation of the gelatin ink yielded a contractile cardiac tissue (approximately 1 cm long) with perfusable vascular channels. In freeform reversible embedding of suspended hydrogels (FRESH) bioprinting, the direct extrusion of ECM proteins and cells within a gelatin microparticulate support bath has enabled the fabrication of various cardiac structures using a type I collagen bioink, including perfusable multiscale vasculature (Fig. 2B), a tri-leaflet heart valve, and a neonatal-scale scale human heart (Fig. 2C).^5^ A full-size model of an adult human heart was also FRESH-printed for surgical practice using an alginate ink (Fig. 2D).^26^ These examples illustrate bioprinting control of microscale and macroscale structure.

In terms of function, 3D bioprinting has enabled the engineering of centimeter-scale tissue constructs that few molding, electrospinning, or other approaches have been able to attain. This builds on earlier work in the field of cardiac tissue engineering,^8^ including electromechanical conditioning to achieve more mature function.^34^ The importance of structure–function relationships has been reinforced through engineered ventricular heart chambers^35^ and electrospun ventricular chambers with controlled helical alignment of cardiomyocytes^36^ that achieve measurable pump function. 3D bioprinted constructs build off the lessons learned in these previous studies. For example, SWIFT-printed cardiac tissues (approximately 6 mm wide) exhibited contractility, with sensitivity to electrical pacing (Fig. 2E).^31^ FRESH 3D bioprinting has been used to create a range of cardiac tissues with diverse functions. A simplified model of a left ventricle (8 mm base to apex) was FRESH-printed using a type I collagen bioink and a fibrinogen-based bioink with hESC-derived cardiomyocytes and fibroblasts.^5^ These ventricles had circumferential calcium wavefront propagation in the print direction, demonstrated wall thickening and decrease in chamber volume when field stimulated to synchronously contract, and could manifest complex arrhythmias under specific conditions (Fig. 2F). Contractility was also demonstrated in a FRESH-printed linear heart tube (13.5 mm in length), which was created by casting a collagen gel with hESC-derived cardiomyocytes and fibroblasts around a bioprinted collagen tube (Fig. 2G).^32^ Pumping resulted in unidirectional displacement of fluorescent beads from within the lumen. Additionally, FRESH was also used to engineer human chambered muscle pumps (hChaMPs) with chambers and large vessels akin to the adult heart (approximately 2 cm long and 1 cm in diameter).^4^ These hChaMPS demonstrated calcium handling, contraction, pumping, sensitivity to pharmacological stimuli, and generation of pressure-volume loops (Fig. 2H). However, hChamps had lower conduction velocities and minimal pumping compared to embryonic and adult hearts. This is the state-of-the-art for engineered heart tissues, which generate only a fraction of the conduction velocity, contractile force, and fractional shortening of adult cardiac tissue. For example, the maximum contractile force reported for engineered heart tissue is only approximately 25% of the force generated by the adult heart.^37^ Nevertheless, engineered heart tissue continues to improve as new advances in tissue engineering are applied to build heart muscle in more complex 3D architectures.

Current preclinical data for bioprinted cardiac tissue are mostly limited to in vitro models; however, a few proof of concept in vivo models have also been explored. For example, Gaetinini et al implanted a small, 3D-printed cardiac patch, consisting of a hyaluronic acid/gelatin scaffold with human cardiomyocyte progenitor cells (CMPCs), into a myocardial infarction model in non-obese diabetic (NOD) severe combined immunodeficient (SCID) mice to prevent rejection of the human cells. The patch-maintained cell viability for up to 1-month post-implantation and significantly reduced end diastolic volume and end systolic volume compared to the no treatment control, suggesting enhanced cardiac function.^33^ Beljeri et al also showed promise with this approach by implanting a 3D-printed disc containing human CMPCs, decellularized cardiac ECM, and gelatin methacrylate into the epicardium of a rat. The disc was retained for up to 14 days in vivo and histological analysis of the explant showed vascular ingrowth.^38^ Although both studies used relatively simple geometries for their printed constructs, they provide proof of concept of the potential for 3D bioprinting to improve cell engraftment, cardiac function, and vascularization. Future in vivo work should focus on leveraging the unique advantages of 3D printing to better recapitulate the structure-function relationships observed in cardiac tissue.

## Ongoing Challenges in 3D Bioprinting Cardiac Tissue

### Manufacturing Billions of Cardiomyocytes with the Required Phenotype

Pluripotent stem cells can be differentiated into many cell types within your body, including cardiomyocytes and most other cardiac cell types,^3^ but there are limitations in scaling up production to generate the billions of cells required for the whole heart. If we focus on cardiomyocytes, it is estimated that approximately 1 billion cardiomyocytes are needed to repair a myocardial infarction that destroys 25% of the left ventricle.^39^ This number increases when we consider building a whole heart, where it is estimated that 3-4 billion cardiomyocytes are needed,^40^ in addition to other cell types (eg, fibroblasts, endothelial cells, smooth muscle cells, nodal cells, and Purkinje cells). It is challenging to create this number of cells using standard 2D culture and differentiation of hIPSCs, where ~400 T75 flasks with a yield of ~10 × 10^6^ cardiomyocytes each would be needed to build a single heart. This is a labor-intensive task requiring hundreds of person-hours, combined with an estimated cost of reagents and plasticware for stem cell expansion and differentiation of ~$170 per T75 flask of cardiomyocytes. When adding in the labor required to produce these cells by a trained scientist, it is estimated to cost >$130 000 to generate the billions of cardiomyocytes required to 3D bioprint a single, adult-sized human heart. If we also consider the generation of other cell types, such as fibroblasts, endothelial, and cardiac conduction system cells, this will increase costs substantially.

**Figure 3.**
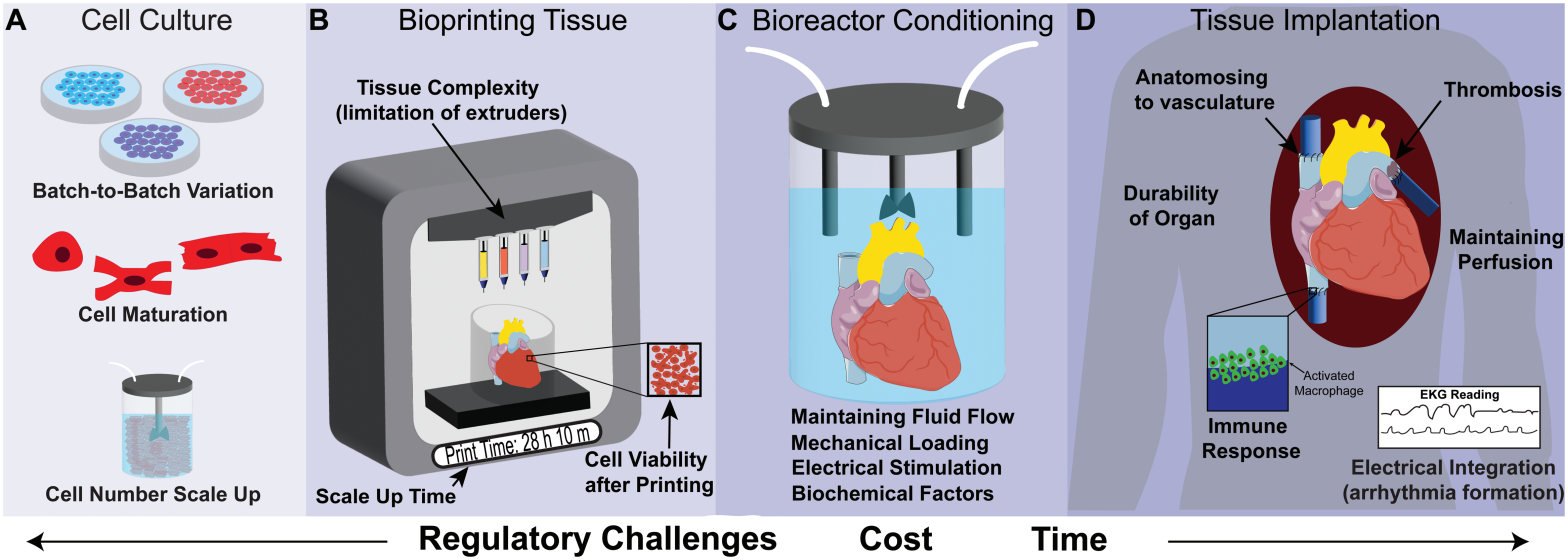
Challenges to bioprinting cardiac tissue for transplantation. (A) In the cell culture phase, phenotypically immature cardiomyocytes and batch-to-batch variations in differentiation efficiency and quality can affect cell function. (**B**) In the 3D bioprinting phase, organ-scale constructs require long print times and extrusion can impart shear stress on the bioink, both of which can decrease cell viability. Further, the different tissue types in the heart (eg, myocardium, conduction system, valves, etc.) adds complexity in terms of the number of bioinks needed and therefore the number of extruders on the printer. (**C**) In the post-printing phase, tissue culture bioreactors are required to mimic in vivo-like mechanical loading, electrical stimulation, and/or biochemical factors to mature the tissue. (**D**) In the in vivo implantation phase, specialized surgical procedures to anastomose the engineered construct to host vasculature and/or post-operative management of arrhythmias may also be needed.

Many approaches are being investigated to scale hiPSC-derived cardiomyocyte production. Multilayer culture plates^41^ and polydimethylsiloxane-lined roller bottles^42^ have enabled the batch production of > 1 billion cardiomyocytes; however, yield is still limited by the surface area. Expansion of cardiomyocytes up to 250 times post differentiation using CHIR 99021 is also possible^43^ but requires low cell density and passaging multiple times to achieve, and questions about functional changes remain. Conditional immortalization of cardiomyocytes has also been shown, resulting in doxycycline-controlled expansion and subsequent redifferentiation,^44^ but it is unlikely that viral oncogene insertion would be viable for clinical translation. Alternatively, suspension bioreactor 3D expansion and differentiation of hiPSCs into cardiomyocytes has the potential to generate the billions of cells required to build an entire heart.^45^ An advantage of bioreactors is that sensors can be used in real time to monitor and control culture conditions, minimizing batch-to-batch variability (Fig. 3A). Several groups have cultured hiPSC aggregates in suspension bioreactors and differentiated them into multiple lineages. For example, Ho et al used bioreactor culture of pluripotent and differentiated cells and bioprinted them into various structures.^46^ However, to date there has been minimal work comparing the structural and functional maturity of cardiomyocytes generated under different protocols (2D versus 3D, small molecule versus growth factor-based).

Cost is also a major concern, where several groups have investigated the minimum number of essential reagents required to maintain stem cell pluripotency^47-50^ and subsequent differentiation into cardiomyocytes.^51,52^ Recent work has also optimized the formulation of specific recombinant proteins, the primary cost drivers in stem cell media, resulting in a reduction in cost to ~3% of commercially available medias, and also allows for weekend-free culture.^53^ Future work will be needed to focus on how effective these cost-saving media formulations and protocols are in terms of functional differences in the resulting differentiated cells.

Finally, any transplanted tissue needs to avoid immune rejection in the patient in order to achieve long-term in vivo function. One option is patient-specific hiPSCs to eliminate the need for immunosuppression; however, it is a time-consuming and expensive process to generate clinical-quality hiPSCs and differentiated cardiomyocytes on a per patient basis. An alternative option is allogeneic hiPSCs lines optimized for 3D bioreactor expansion and differentiation as a scalable and economical solution. Here the concern would be immune rejection due to unrecognized antigens such as human leukocyte antigen (HLA) on the surface of cells.^54^ In this case, systemic immune suppression similar to what is currently used for organ transplantation is possible, and perhaps realistic for a first generation of 3D bioprinted heart tissue. However, systemic immune suppression has a number of side effects and only slows grafts rejection rather than preventing it. To address this broadly, a number of researchers and companies are developing hypoimmune hiPSCs that can be manufactured at scale under good manufacturing practices (GMP) conditions and serve as universal donor cells. For example, using CRISPR-Cas9 gene editing to create hiPSCs with reduced immunogenicity that do not express any HLA class I or II on their surface.^55-57^ Unfortunately, removing specific HLA genes may not be a definitive solution as natural killer cells will attack cells that lack HLA on their surface.^58^ This is just one example of immune tolerant hiPSCs being developed and highlights the need for continued development of strategies to prevent immune rejection in cell and engineered tissue-based therapies.

### Developing Cell-Laden Bioinks Optimized for Tissue Formation

The combination of cells and typically some form of biomaterial is termed the bioink. The composition and properties of the bioink are critical in obtaining functional bioprinted heart tissue.^59-61^ A key research area is identifying the cellular building blocks to incorporate into the bioink, either single cells, multi-cellular aggregates, or organoids. Here, organoids are defined as self-organized, 3D multicellular structures that recapitulate certain features of the full organ.^62^ For example, cardiac organoids can form ventricle-like cavities and vascular structures reminiscent of embryonic heart development, and one could imagine printing these to form more complex tissue. Although work suggests that these organoids can fuse at early stages,^63^ gaps remain in our understanding of how these cells self-assemble and is untested in the context of bioprinting. A clear disadvantage of printing cardiac organoids or aggregates is their size, which typically ranges from 150 to 500 µm in diameter, which limits resolution. Specifically, the inner diameter of the needle used to extrude a cellularized bioink is typically 2-3× the cell aggregate/organoid size in order to minimize shear stress on the cells, so in this case 300-1500 µm. In contrast, single cells that have a diameter of 20-30 µm can be used for bioinks with a needle inner diameter of 50-100 µm, improving resolution of the printed structure.^64^ However, the solution does not need to be single cells or aggregates, as multi-extruder 3D bioprinters^65,66^ can print both bioinks; however, the downside of multiple extruders is the extra time required to print due to switching between the nozzles (Fig. 3B). Given the size and complexity required to print a heart, it is also likely that whole hearts would take a significant time to print, which could potentially impact cell viability within the construct. For example, a full-scale human heart printed using alginate took approximately 92 hours to complete.^26^ Future studies will be needed to determine the minimum resolution, number of bioinks required to recreate tissue complexity, and the relationship between cell viability and print time.

A related question is the required maturation state of the printed cells and if it is advantageous to print immature cells and differentiate and/or mature them post-printing, or to do so prior to the printing process. Several studies demonstrate that immature cardiomyocytes have increased attachment in myocardial infarction models,^3^ better survival under adverse conditions (such a cryostorage^3^), improved proliferation,^67^ and in the case of second heart field progenitors, migration toward damaged heart muscle to repair it.^68^ Immature cardiomyocytes may also be more responsive to both electrical and mechanical stimuli, facilitating maturation.^34^ In contrast, mature cardiomyocytes contribute more to the structure and function of the tissue following integration, specifically improved myofibril organization and greater improvements in contractile and electrophysiological function.^3,42^ Mature cardiomyocytes also demonstrate a reduction in automaticity and subsequent complications such as arrhythmias,^42^ which is an important consideration. Pluripotent stem cells can also be printed directly and subsequently differentiated into cardiomyocytes post-printing. This has been used to build ventricle-like chambers, although there was spatial variability in cardiomyocyte density due to diffusion gradients that impacted both differentiation and viability.^4^ In situ differentiation post-printing is also limited in the types of cells that can be differentiated due to the exact differentiation media used, which is often cell type specific.

### Post-Print Tissue Maturation and Function

Physiologic conditioning and maturation at both the cellular and tissue levels are also necessary post-printing to engineer tissue with the required structure and function. For example, cardiac tissue is composed of aligned cardiomyocytes connected end-to-end via intercalated discs^69^ that are necessary for the exchange of ions and small molecules as well as electro-mechanical coupling.^70,71^ In a bioink, the cells are typically in suspension and thus have a spherical morphology. To achieve organization in a bioprinted tissue, cardiomyocytes must spread out within the printed construct, elongating to recover polarity and reforming cell-cell junctions to form a syncytium of highly aligned myofibers. Current hiPSC-derived engineered heart tissue exhibits immature structure and function, including decreased myofibrillar density and alignment, contractility, and conduction velocity, when compared to native heart muscle.^72,73^ Some key features of adult heart muscle tissue include: (1) highly aligned myofibrils, (2) contractile stresses of 15-30 kPa, 3) conduction velocities of 30-100 cm/s, (4) action potential upstroke velocities of 150-350 V/s, (5) formations of a sarcoplasmic reticulum and t-tubule network, and (6) the presence of intercalated discs.^74,75^

To improve this, maturation strategies aim to recapitulate the native microenvironment by incorporating multiple cell types (including fibroblasts and endothelial cells) and utilizing biochemical, electrical, spatial, and/or biomechanical cues.^75–77^ For instance, fibroblasts and endothelial cells can interact with cardiomyocytes via gap junctions to regulate electromechanical signaling or establish connections to vasculature, respectively.^78^ Small molecules and growth factors can be used to modulate signaling pathways to drive stem cell differentiation into cardiomyocytes and subsequent maturation, as well as drive cellular processes like vascularization.^79^ Electrical stimulation, which aims to mimic the beat rates found in vivo, have produced cardiac tissues with more mature electrophysiology.^34,80^ Topographical and spatial patterning of cells have also been used to direct uniaxial cardiomyocyte alignment, which is required to achieve maximum contractile force.^30,81–83^ Tissue-scale microarray analysis and single-cell transcriptomics further support the finding that maturation can be improved in 3D engineered cardiac tissue.^34,84,85^ Finally, physical conditioning that mimics the biomechanical forces in the ventricular wall of the heart, such as preload and afterload, has also been shown to improve contractility and produce and more mature cardiomyocyte phenotype.^34,80,86,87^ Combining multiple maturation strategies together into an integrated bioreactor culture system is ongoing work in the field, with the goal to understand the parameters needed to engineer cardiac tissue with adult-like function that will ultimately be required for transplantation.

Bioreactor-based systems for the maturation of cardiac tissues are promising because they can recapitulate multiple aspects of the native environment (Fig. 3C). Electrical stimulation and mechanical conditioning via tissue stretching or compression has been incorporated into some bioreactors.^79^ Perfusion bioreactors are particularly exciting because they improve oxygenation and mimic biomechanical forces, such as changes in blood pressure and volume experienced in vivo.^88^ Multiple strategies can be combined into a single bioreactor platform, such as mimicking 2 aspects of the native microenvironment: (1) passive tension by casting around elastomeric pillars and (2) electrical stimulation.^34,80^ Together, this generated more mature cardiomyocytes and tissues that produced more force than human fetal cardiac tissue, although still significantly lower than adult myocardium. This demonstrates the use of an electrical stimulation regimen to mature cardiomyocytes, although to date this approach has not been applied to 3D bioprinted cardiac tissue, likely due to the more complex bioreactor required.

Advances in stem cells, 3D bioprinting, and bioreactors have the potential to be integrated into a manufacturing process for the biofabrication of transplant-grade organs. This could potentially involve an on-demand process via the following steps: (1) generating patient-specific hiPSCs, (2) hiPSC expansion and differentiation of all cell types needed, (3) organ fabrication via 3D bioprinting using patient MRI data to inform organ dimensions, (4) bioreactor perfusion and electromechanical conditioning and maturation, and (5) transplantation in the patient. Alternatively, hypoimmunogenic hiPSCs could be used in place of patient-specific hiPSCs, meaning hearts could be biofabricated at scale and be available as an off-the-shelf option. Regardless of cell source, both approaches are still at the early research stage, with many challenges to overcome before such technology becomes a clinical option.

## Conclusions and Future Directions

Biofabrication of a human heart as a viable alternative to transplantation has remained a moonshot for the tissue engineering field for decades. In this concise review, we have covered major progress toward this goal, including the use of pluripotent stem cells differentiated into cardiomyocytes and other cell types, combined with an advanced biomanufacturing technology such as 3D bioprinting to create the required 3D structure. There have been many important advances in the field showing that functional 3D cardiac tissues can be bioprinted into relatively advanced, ventricle-like structures that can achieve hallmarks of organ function such as wall thickening and pressure-volume loops.^4,5^ Bioreactors have also demonstrated the potential for both the culture and scale-up of progenitor and differentiated cardiac cells, as well as the maturation and physiologic conditioning of 3D bioprinted constructs. However, the contractility does not match native tissue, so it is also clear that further development is needed to engineer cardiac tissue with the structure and function required for clinical translation.^87^ In fact, only a single phase I clinical trial is in progress at the time this review was written that includes the implantation of engineered heart muscle tissue with hiPSCs for heart failure.^89^ Instead, the majority of clinical trials aimed at regenerating heart tissue rely on the injection of a patient’s own adult stem cells, including mesenchymal stem cells and cardiac progenitor cells. While over 120 such trials have been completed or are currently active, their clinical efficacy has been limited due in large part to poor engraftment and survival of the implanted cells.^87,90,91^

While transplantation of engineered heart tissue has yet to reach the clinical trial stage, significant progress has been made in the development of engineered heart tissue as physiologic and pathophysiologic models for drug discovery. Here, the advantage over current microphysiologic systems is the ability of 3D engineered heart tissue to more closely mimic physiologic contractility, including ventricular pump function.^92^ For example, Costa and coworkers engineered a human ventricle-like cardiac organoid chamber (hvCOC) using a molding approach that demonstrated dose-response to inotropic agents and other pharmacological compounds.^35^ Radisic et al implemented a different molding approach to engineer a 3D cardiac tissue with distinct atrial and ventricular tissues using healthy and hypertensive hiPSCs to model left ventricular hypertrophy.^93^ Various other approaches have also been developed for studying human cardiac disease using 3D molded tissues,^94^ organoids,^95,96^ and microfluidics.^97,98^ Only recently 3D bioprinted cardiac models for in vitro pharmacology have been demonstrated, such as the collaboration between FluidForm and Merck using FRESH 3D bioprinting to create highly aligned and dense cardiac tissues that show inotropic, chronotropic, and myotropic responses.^99^ It is expected that the quality and predictive ability of in vitro engineered heart tissue will continue to improve due to government policies such as the Food and Drug Administration (FDA) Modernization Act 2.0, which is working to reduce animal testing while improving the translational relevance of data from in vitro systems.

To our knowledge, there are currently no clinical trials focusing on heart regeneration with a 3D bioprinted cardiac tissue, and the FDA has yet to approve a cellularized 3D bioprinted tissue of any type for any indication. To get to this point, the field will need to address key criteria for engineering a whole heart, including those beyond the biofabrication stage, such as in vivo implantation and engraftment (Fig. 3D). Vascular integration between the host and tissue will need to be accomplished, which is commonly done by anastomosing the patient’s vessels to the engineered tissue graft. This can be a complicated surgery and requires highly trained cardiothoracic surgeons. Potential surgical complications include thrombosis formation at the anastomoses site or within the vasculature of the engineered graft itself.^100^ Another major concern is the immune response post-implantation due to both the surgery itself and the constituent cells. Although immunosuppression, patient-specific cells, or haplotype matching can be used to diminish the immune response, each of these approaches has inherent limitations. Electrical integration is also imperative to achieve electromechanical coupling between the 3D bioprinted construct and the existing heart muscle; otherwise, deadly arrhythmias could arise. Finally, organ durability in vivo will need to be established since the cardiac tissue graft will experience physical manipulation and suturing during implantation, and exposure to physiologic blood flow and pressures post-implantation. Vascular grafts typically need to withstand a burst pressure of 2000 mmHg as a safety margin for clinical use,^101^ an order of magnitude more than normal systolic pressures of approximately 120 mmHg, setting a high bar for cardiac tissue grafts to achieve. Looking forward, it will be necessary to continue to push the boundaries of stem cell biology and additive manufacturing to enable the biofabrication of cardiac muscle tissue with the hierarchical structure, necessary cell types, and ECM to produce the desired contractile function required for heart repair, regeneration, or replacement.

## Data Availability

No new data were generated or analyzed in support of this research.
